# Mapping the spread of fluoroquinolone resistance: continued presence of non-susceptible *Escherichia coli* in broilers

**DOI:** 10.3389/fvets.2025.1610997

**Published:** 2025-09-19

**Authors:** Moniek Ringenier, Filip Boyen, Bert Bogaerts, Mathieu Gand, Kevin Vanneste, Sigrid C. J. De Keersmaecker, Ilias Chantziaras, Nelima Ibrahim, Mathias Devreese, Jeroen Dewulf

**Affiliations:** ^1^Department of Internal Medicine, Reproduction and Population Medicine, Faculty of Veterinary Medicine, Ghent University, Merelbeke, Belgium; ^2^Department of Pathobiology, Pharmacology and Zoological Medicine, Faculty of Veterinary Medicine, Ghent University, Merelbeke, Belgium; ^3^Transversal Activities in Applied Genomics, Sciensano, Brussels, Belgium

**Keywords:** fluoroquinolone, *Escherichia coli*, poultry, antimicrobial resistance, whole genome sequencing, antimicrobial usage

## Abstract

**Introduction:**

Sustained resistance against fluoroquinolones (FQ), without the use of FQ in broilers, raises important questions about other possible factors contributing to the persistence in farms. Therefore, the current study investigates the prevalence of FQ non-susceptibility in *Escherichia coli* on broiler farms and examines the roles of day-old chicks, the farm environment, and the antimicrobial use (AMU) in the dynamics of the within-flock spread.

**Methods:**

On 29 Belgian broiler farms, AMU was monitored, while environmental and day-old chick intestinal samples (day 0) were collected before their arrival. On days 3 and 36 of the production round, 30 cloacal swabs were taken per farm. In all samples, total *E. coli* and FQ non susceptible *E. coli* isolates were quantified by non-selective and FQ selective isolation. A selection of the isolates was analyzed using whole-genome sequencing to characterize their resistance and virulence-associated determinants and to investigate their phylogenetic relatedness using core-genome multi-locus sequence typing (cgMLST) and whole-genome single-nucleotide polymorphism (SNP) analysis.

**Results:**

Before entering the stable, day-old chicks carried FQ non-susceptible *E. coli* on 79.3% of the farms, while FQ non-susceptible *E. coli* were found in 48.3% of the sampled environments. According to cgMLST, identical FQ non-susceptible isolates were found on day 0 and 36, suggesting that FQ non-susceptible isolates present in the environment at the start of a production round or in day-old chicks, can remain present until slaughter, even when no FQs were used. Total AMU was positively correlated with the proportion of FQ non-susceptible *E. coli* [*r* = 0.42, 95% CI (0.06, 0.68), *p* = 0.03], often also multidrug-resistant, at the end of the production cycle.

**Conclusion:**

The continued presence of FQ non-susceptible *E. coli* in broiler farms is likely the result of both a historical contamination at the farm level and a continuous influx along the production chain. AMU contributes to the continued presence of FQ non-susceptible *E. coli* in broiler farms, but accounted for only a small proportion of the variability in FQ non-susceptibility in currently investigated farms. The role of certain virulence-associated genes in the persistence of FQ non-susceptible *E. coli* in broiler farms deserves more in-depth research.

## 1 Introduction

Antimicrobial resistance (AMR) is of critical concern for both human and animal health. The emergence of AMR is a complex issue with antimicrobial usage (AMU) often being the main driver (1). Several studies have demonstrated a link between lowering AMU and a reduction in AMR in food producing animals (2–4). However, in countries or farms not using any (fluoro)quinolones anymore, quinolone resistance in *Escherichia coli* has been shown to persist (5–8).

The sustained resistance against fluoroquinolones (FQ), without the use of FQ in broilers, raises important questions about possible other factors contributing to the persistence in these farms. Several studies showed that day-old chicks and the broiler house environment are involved in the introduction and transmission of extended-spectrum *β*-lactamase (ESBL) producing *E. coli* in broiler flocks (9–13). For instance, Huijbers et al. (3) demonstrated that both introduction routes contributed to flock colonization, where nearly all broilers tested positive for ESBL-producing *E. coli* 3 days after the start of the production cycle. Also Dierickx et al. (10) confirmed this rapid spread within a broiler flock, and was able to link isolates from day-old chicks to isolates in parent stock, suggesting a transmission route from breeding flocks to hatcheries and subsequent uptake by chicks from the hatchery environment. This form of pseudo-vertical transmission in the hatchery was also described by Projahn et al. (12). On top of that, environmental recirculation of resistant strains from previous production cycles was suggested, as resistant strains were detected even after cleaning and disinfection, a finding also supported by Daehre et al. (13). Furthermore, insects have been identified as potential vectors contributing to the introduction and dissemination of ESBL-producing *E. coli* within broiler houses (11).

As far as FQ resistance is concerned, several studies suggest vertical transmission of FQ resistant *E. coli* within the broiler production pyramid, highlighting the potential role of breeding animals (8, 14–17). This has been demonstrated by linking isolates from (grand)parent stock to broilers samples retrieved at the end of the production cycle (8, 14–17). Notably, these studies often focussed on sampling at the end of the broiler production cycle and often did not investigate transmission within a flock during the production cycle and the contribution of other potential risk factors such as AMU at the farm level. Petersen et al. (16) was able to link FQ resistant *E. coli* in parent stock to hatchery fluff and hatching eggs causing an outbreak of colibacillosis in broiler flocks, indicating vertical transmission. Also, several studies highlighted the role of the environment in the circulation of FQ resistant *E. coli* among broilers (14, 15, 18, 19). Börjesson et al. (15) could trace strains from grandparent bird to broilers and found resistant *E. coli* with identical patterns in hatcheries and poultry houses, suggesting environmental involvement. However, no direct link was visible between broiler environment and the housed broiler batch. This persistence in the broiler house environment was also suggested by Kaspersen et al. (14). In an experimental setting, Leclerq et al. (20) observed that multi-drug-resistant *E. coli* could survive in the environment and potentially recirculate. However, they found no evidence of introduction of resistant strains through day-old chicks. The study did emphasize the need for research under commercial farm conditions, where biosecurity measures would presumably be less controlled than in experimental environments. While several introduction and persistence routes for FQ-resistant *E. coli* in broiler production have been proposed, including vertical transmission and environmental contamination, most existing studies have examined these factors in isolation. Large, comprehensive studies that simultaneously take into account the role of the environment, day-old chicks and AMU, are lacking. To effectively combat FQ resistance in the broiler industry, a more holistic understanding of its presence and dissemination within broiler flocks is essential, allowing for targeted interventions to reduce its prevalence.

The purpose of this study was to investigate the role of AMU, day-old chicks and the environment in the spread of FQ non-susceptible *E. coli* within broiler flocks. Furthermore, the goal was to see how this FQ resistance persists throughout the production cycle and what the (genetic) AMR traits are of the persisting non-susceptible *E. coli* strains.

## 2 Materials and methods

Approval for this study was granted by the ethical committee of the Faculties of Veterinary Medicine and Bioscience engineering of Ghent University (EC2020/040).

### 2.1 Farm characteristics

This study included 29 commercial broiler farms from the Flanders region of Belgium. Farms were included based on convenience sampling, as they were recruited through outreach efforts facilitated by local veterinary poultry practices. Participation was on a voluntary basis and only conventional broiler (Ross 308) farms in Belgium were included. Per farm, one house was randomly selected for follow-up during one production cycle and samples were collected to determine prevalence of FQ non-susceptible *E. coli* on day 0, 3 ± 2 days, and 36 ± 3 days of that production round. Also, information was collected on farm size, hatchery, age of the parent stock, and AMU during that specific production round. AMU of the specific production cycle was collected by a form which was filled in by the farmer during the sampling period. Quantification of AMU was done through the calculation of the Treatment Incidence (TI) per 100 days, as described by Persoons et al. (20, 21). The TI is equal to the number of days animals are treated with antimicrobials in a theoretical period of 100 days. Sampling was conducted between October 2020 and December 2021.

### 2.2 Sample collection environment

Environmental swabs from the stable were collected on day 0 before the day-old chicks arrived, when bedding material was already placed and the feeding pans were filled. For the environmental swabs premoistened sponge swabs with 10 mL Buffered Pepton Water (BPW) were used (3 M, SLL10BPW, St-Paul, United States). Five locations were sampled in duplicate: the floor and boots in the anteroom, and the floor, drinking cups, and feeding pans in the house. An A4-sized floor area was sampled per swab, while three drinking cups, one feeding pan per swab, or both soles of a pair of boots were sampled per swab. These locations were chosen based on previous research, which identified these locations as high risk areas for contamination with bacteria, even after cleaning and disinfection (22). Also, drinking water samples were collected at the end of the drinking line in each house after cleaning the line with a disinfectant and running the water for around 3 min. The sample was sent to an external laboratory for bacteriological analysis.

### 2.3 Sample collection broilers

On day 0, 3, and 36, chicks were sampled to screen for gastrointestinal carriage of FQ non-susceptible *E. coli*. On day 0, 30 day-old chicks were randomly collected before they were unloaded in the house. The chicks were transported to the laboratory in a cardboard transport box, followed by euthanasia through cervical dislocation and collection of the entire gastrointestinal tract and contents. On day 3 and 36, cloacal samples were collected from 30 randomly selected chicks using sterile rayon-tipped swabs (Vacutest Kima, Italy). Also, on day 36, three broilers were euthanized and feather samples were collected to screen for FQ residues. All collected samples were cooled directly, transported at a temperature of 2–7°C and processed within 4 h after collection.

### 2.4 FQ residues in feathers

To detect residues of FQ in the feathers of broilers, a validated UPHLC-MS/MS method was used (23). The feathers were screened for the presence of ciprofloxacin, enrofloxacin and flumequine residues. The residue concentration of the complete feather sample was determined by weighing around 500 mg of feathers, followed by cutting the feathers into smaller pieces and grinding. From every grinded feather sample, around 100 ± 1 mg was weighed and further processing took place as described in Ringenier et al. (23). The limit of quantification (LOQ) of the used method was 5 ng/g feather for enrofloxacin and ciprofloxacin, and 1 ng/g feather for flumequine.

### 2.5 Bacteriological analysis

In the day-old chicks, the entire gastrointestinal tracts were removed with sterile material, cut into smaller pieces, weighed and transferred into a stomacher bag. Subsequently, sterile BPW (Oxoid, Thermo Fisher Scientific, Merelbeke, Belgium) was added (in ratio of 9 mL of BPW per gram of intestines) and thoroughly mixed using a stomacher. Next, 100 μL of the mixture was inoculated on FQ selective (MacConkey agar Nr 3 (Oxoid, Thermo Fisher Scientific, Merelbeke, Belgium) supplemented with 0.25 mg/L enrofloxacin) and FQ non-selective (MacConkey agar Nr 3) media. These plates were aerobically incubated overnight at 37°C, as well as the intestine suspensions in BPW. If no growth was visible on the (supplemented) MacConkey agar plates, 100 μL of the enriched broth was inoculated on a MacConkey plate and/or a MacConkey plate supplemented with 0.25 mg/L enrofloxacin. The concentration of enrofloxacin in the supplemented plates was used to select for non-wild type isolates (0.25 mg/L is the epidemiological cut-off value (ECOFF) for enrofloxacin in *E. coli*). “FQ non-susceptible *E. coli* isolates” will be used in this article for FQ non-wild type isolates.

To the environmental swabs, 10 mL of sterile BPW was added immediately after transportation to the laboratory. After homogenization, using a stomacher, the swab suspensions were aerobically incubated for 24 h at 37°C. After incubation, 10 μL of this suspension was plated on FQ selective and FQ-non-selective media as described above and aerobically incubated overnight at 37°C.

One hundred milliliter of the collected water samples was filtered through a 0.45 μm filter. The filter was placed on non-supplemented Rapid *E. coli* (Bio-Rad) agar plates and Rapid *E. coli* agar plates supplemented with 0.25 mg/L enrofloxacin to screen for FQ non-susceptible *E. coli*. Plates were aerobically incubated at 37°C for 24 h. The colonies were counted and the number of *E. coli* isolates was determined per 100 mL.

Cloacal swabs were weighed before and after collecting feces to determine the amount of feces collected. Three milliliter of BPW was added and the swab was thoroughly vortexed. Next, 100 μL of the feces suspension was used to make 10 fold serial dilutions (from 10^−1^ to 10^−4^) in Phosphate Buffered Salt solution (PBS). Of every dilution, 100 μL was inoculated on FQ selective and FQ non-selective media. These plates were aerobically incubated overnight at 37°C, as were the swabs containing BPW broth for microbial enrichment. The concentration of FQ non-susceptible *E. coli* and total *E. coli* in the cloacal samples were determined (CFU/gram feces) based on the counts on plates with 20–200 CFU and taking into account the weight of the feces on the swab (ranging between 0.01 and 0.34 gram). The proportion of CFU grown on FQ selective over FQ non-selective MacConkey plates allowed to determine the prevalence of FQ non-susceptible *E. coli* strains in a specific cloacal sample. Identification of the isolates at the species level were done by MALDI-TOF mass spectrometry, as previously described in Vereecke et al. (24). When no growth was visible on the FQ selective or the non-selective MacConkey plates, 100 μL of the overnight enriched broth was inoculated on a second MacConkey plate (supplemented with enrofloxacin) and aerobically incubated overnight at 37°C. When colonies were detected after enrichment only, it was assumed that the concentration was below the detection limit of direct plating. For analysis, the value of such samples was set at the detection limit (for day-old chicks this was 10 CFU/mL BPW and for cloacal swabs 100 CFU/mL BPW) and corrected for the amount of feces on the swabs, as described previously (25).

Of every farm from each sampling point, around 10 randomly selected FQ non-susceptible isolates originating from 10 different animal samples were stored at −20°C. From each positive environmental sample, one FQ non-susceptible *E. coli* isolate was stored. Due to practical and financial constraints, only a selection of isolates was further analyzed.

### 2.6 MIC

For the determination of the minimum inhibitory concentration (MIC), five farms were included based on the results of the prevalence of FQ non-susceptible *E. coli* in the environment and in day-old chicks. Only isolates of day 0 and 36 were included for MIC determination, which resulted in a selection of 92 *E. coli* isolates obtained from FQ supplemented plates. Antimicrobial susceptibility testing was performed using a Sensititre EU Surveillance *E. coli* EUVSEC Plate (Trek Diagnostic Systems, Thermofisher Scientific, Merelbeke, Belgium) according to the manufacturer’s guidelines. The protocol was followed as described in Ibrahim et al. (26). In brief, the Sensititre plate was aerobically incubated at 35°C for 18–24 h. The MIC was determined as the lowest concentration at which no visible growth was observed. The quality control strain used was *E. coli* ATCC 25922. The ECOFFs from the European Committee on Antimicrobial Susceptibility Testing were used for interpretation (27).

### 2.7 WGS

Based on MIC results, a further selection of *E. coli* isolates was made for whole genome sequencing (WGS). Isolates with different resistance profiles from the same sample time point within a farm were included to undergo WGS, resulting in a selection of 66 FQ non-susceptible *E. coli* isolates, originating from four farms. DNA extraction was done using the GenElute™ Bacterial Genomic DNA Kit from Sigma-Aldrich (Merck, Overijse, Belgium) following the manufacturer’s protocol. The quantity and purity of the DNA was determined spectrophotometrically using a NanoDrop (Thermo Scientific, Waltham, MA, United States) and the quantity using fluorometry (Qubit 4, Invitrogen, Carlsbad, CA). A DNA-library was made using the Nextera XT library kit (Illumina). Library fragmentation length and library DNA concentration were evaluated using the TapeStation 4,200 with the HS D5000 ScreenTape and Reagent kits (Agilent Technologies, Santa Clara, CA) and a dsDNA HS assay kit for the Qubit 4 fluorometer, respectively. All libraries were sequenced on a MiSeq instrument (Illumina, San Diego, CA) using the MiSeq V3 chemistry, as described by the manufacturer’s protocol, for the production of 2 × 250 bp paired-end reads. Pre-processing and quality control of the sequenced data were performed using the Shiga-toxin producing *E. coli* pipeline described by Bogaerts et al. (28). NCBI AMRFinder+ (v3.10.18) was used to screen the assembled contigs for genes and mutations associated with AMR using the 2021-12-21.1 database. This database includes genomic markers commonly associated with FQ resistance, such as the often plasmid-encoded *qnr* and *qepA* genes, as well as chromosomal mutations in for example *gyrA* and *parC*. Additionally, the assemblies were screened for the presence of virulence-associated genes using blastn v2.6.0 and the VirulenceFinder *E. coli* database (accessed on July 23, 2023). Sequence typing was performed using the MLST and cgMLST schemes obtained from EnteroBase (29) (accessed on July 7th 2024) as described previously (28). Additional SNP-based clustering was performed for sequence types (STs) with multiple isolates using SnapperDB v1.0.6 (30), of which the selected reference genomes are listed in Supplementary Table S1. Isolates were considered to be clusters if their pairwise SNP distances were 5 or less (31).

### 2.8 Validation of WGS-based taxonomic identification for *Escherichia fergusonii* strains

The WGS data analysis workflow includes taxonomic classification of the trimmed reads using Kraken 2 v2.0.7 and a custom database containing all NCBI RefSeq genome entries (database accessed 2nd November 2021) annotated as “complete genome” with accession prefixes NC, NW, AC, NG, NT, NS, and NZ of the following taxonomic groups: archaea, bacteria, fungi, human, protozoa, and viruses (32). For nine strains, the proportion of reads classified to an unexpected species (i.e., not *E. coli*) was greater than 5%, which is the failure threshold enforced in the workflow (28). For these datasets, the majority of reads were classified as *E. fergusonii*. Additional verification was performed by constructing a core genome MLST phylogeny containing all in-house strains, complemented with 50 randomly selected complete *E. coli* and randomly selected complete *E. fergusonii* genomes from NCBI. In the resulting topology, the nine strains classified as *E. fergusonii* by Kraken 2 clustered with the *E. fergusonii* genomes obtained from NCBI, indicating that this taxonomic identification is likely to be correct.

### 2.9 Statistical analysis

For descriptive statistics, IBM SPSS Statistics 29.0 (IBM, New York, United States) was used. To investigate the relationship between the proportion of FQ non-susceptible *E. coli* at the end of the production cycle and total TI of the production cycle, age of the parent stock, capacity of the stable, proportion of positive day-old chicks, and sum of FQ positive environmental locations; a backwards stepwise regression model was conducted, using *p* < 0.1 for inclusion in the model, resulting in a univariable linear regression model. An independent *t*-test was conducted to determine if there was a significant difference in proportion of FQ non-susceptible *E. coli* on day 3 between the flocks that were treated with lincomycin and spectinomycin and the flocks that did not receive such treatment. An independent samples Kruskal-Wallis test was performed to compare the average number of day-old chicks carrying FQ non-susceptible *E. coli* per farm across different hatcheries.

## 3 Results

This study included 29 commercial broiler farms with an average house capacity of 35,453 birds [min 14,400 - max 80,000]. The batches of day-old chicks from all farms originated from a total of seven different hatcheries. The average age of the parent stock was 48.82 weeks (SD ± 12.80). Farm characteristics are provided in Supplementary Table S2.

### 3.1 AMU

**Figure 1:**
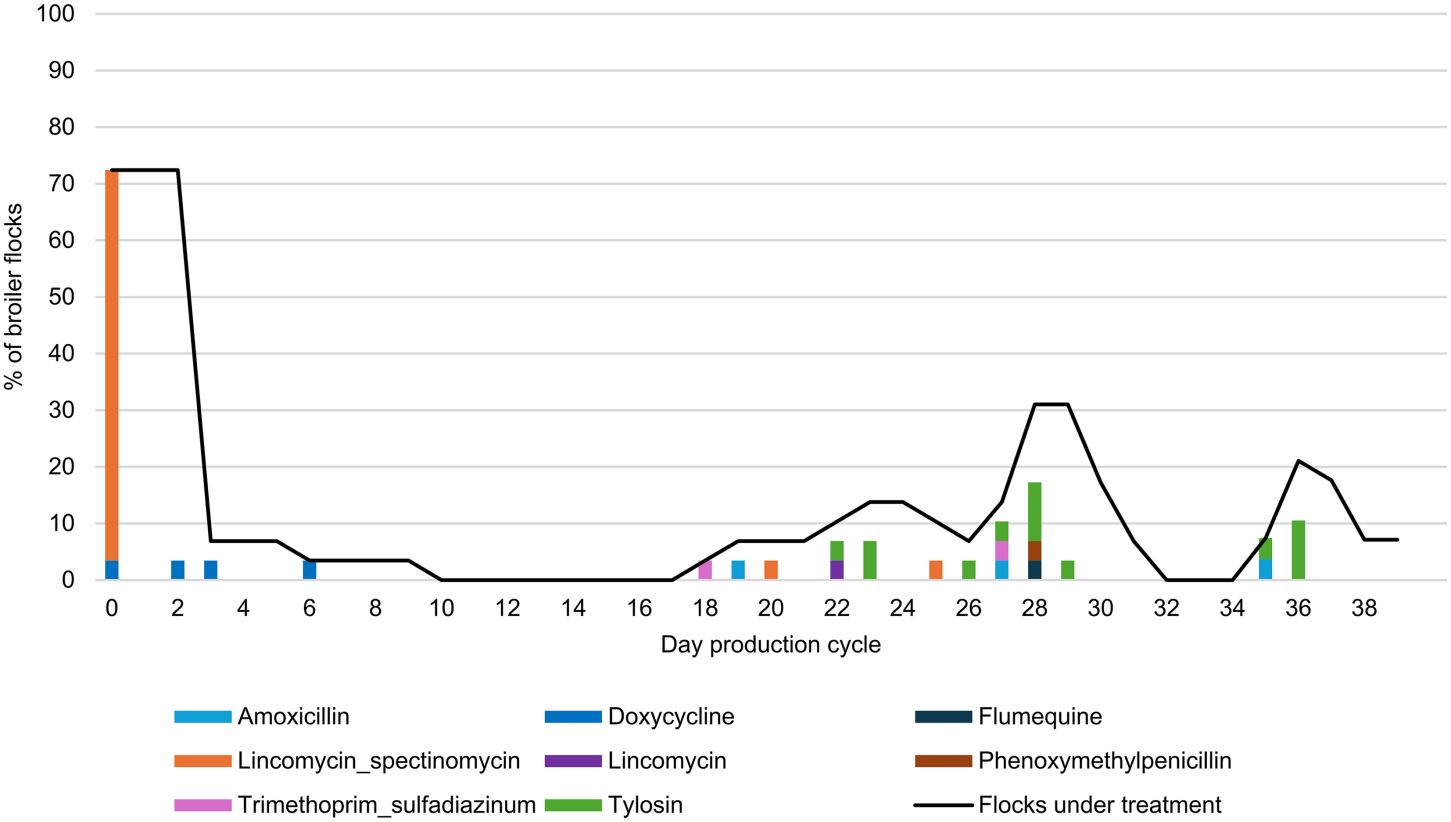
Antimicrobial treatments in the broiler flocks during the observed production cycle (*n* = 29). The results show the percentage of flocks starting treatment per antimicrobial group, together with percentage of flocks under treatment during each day of the production cycle (continuous black line).

On 72.4% (21/29) of the farms, the production cycle started with a course of antimicrobials (Figure 1), of which 95.5% used lincomycin in combination with spectinomycin. Five farms started using antibiotics some days after the start of the production cycle. Only three farms did not administer antimicrobials during the entire production period (Supplementary Table S3). The average TI_DDDvet_ was 4.18 [min 0 - max 33.72], meaning that broilers in this study received antimicrobials during on average 4.18% of their lifespan, which equals to 1.8 treatment days within a production cycle of 42 days. Only one farm reported using a FQ (flumequine) during the observed production cycle. The concentration of flumequine in the feathers from this farm ranged from 50 to 600 ng/g feather (Supplementary Table S4). In the feather samples of the other farms, no FQ concentrations above LOQ could be detected, except for one farm where very low levels of flumequine above LOQ were found, ranging from 1.2 to 3.7 ng/g feather. According to the AMU records, only lincomycin in combination with spectinomycin was administered on this farm in the first 3 days of the production cycle.

### 3.2 FQ non-susceptibility day 0

**Figure 2:**
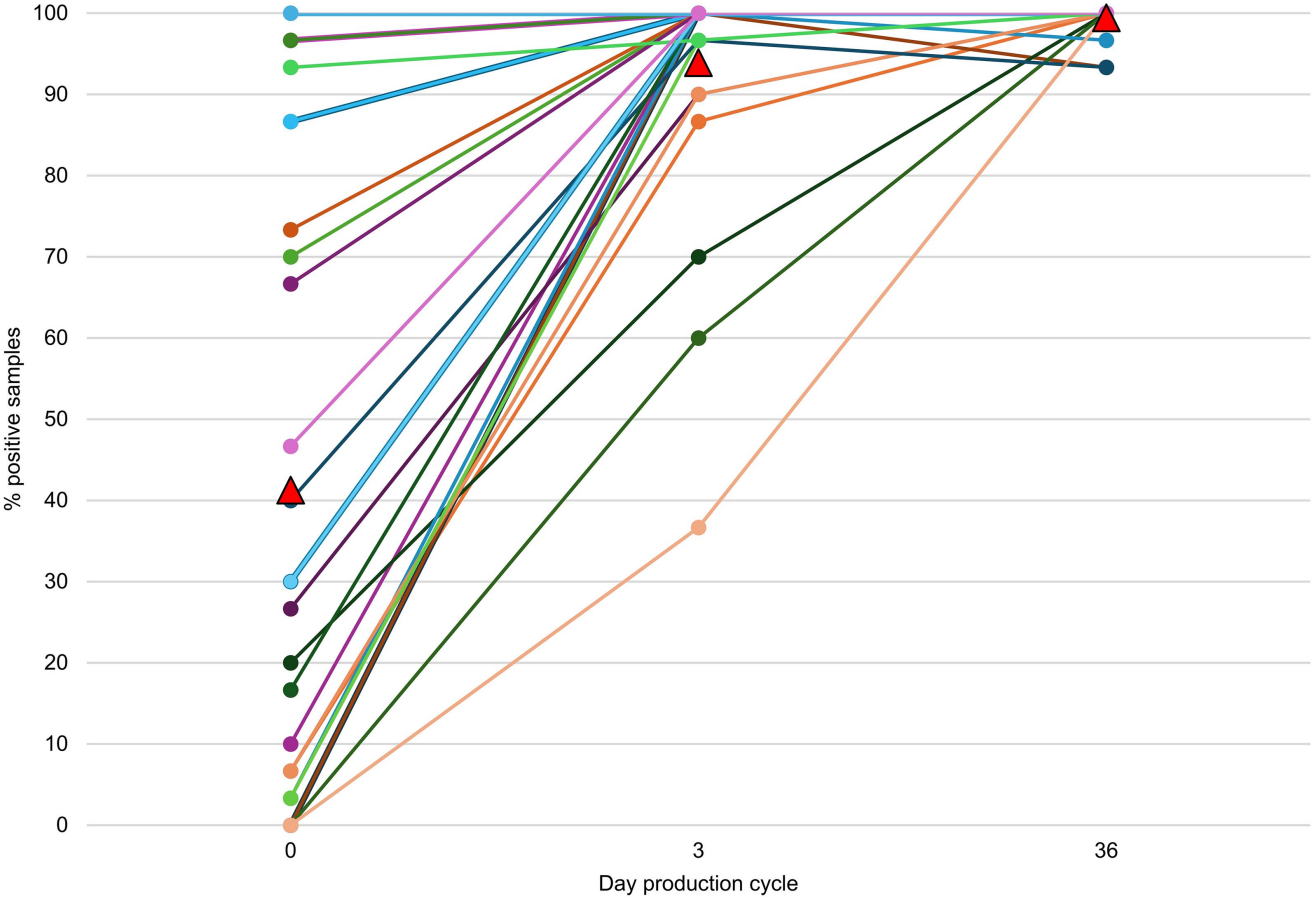
Farm level percentage of samples harboring fluoroquinolone (FQ) non-susceptible *E. coli* per sampling point during one production cycle. On day 0, gastrointestinal contents were sampled and on day 3 (±2) and 36 (±3), cloacal swabs were taken. In six batches of day-old chicks no FQ non-susceptible *E. coli* could be detected. The mean percentage for each time point is depicted as a red triangle.

On 14 farms, at least one FQ non-susceptible *E. coli* isolate could be obtained from the environment before the day-old chicks entered the stable (Table 1; Supplementary Table S5). On 79.3% (23/29) of the farms, FQ non-susceptible *E. coli* were detected in the gastro-intestinal tracts from day-old chicks before entering the stable (Table 2). Of these positive flocks, the percentage of day-old chicks carrying FQ non-susceptible *E. coli* was on average 55.3% [min 6.7 - max 100] (Figure 2). There was no statistically significant difference in the percentage of positive day-old chicks between the different hatcheries (*p* = 0.38), indicating that there was no specific hatchery providing a significant higher or lower number of day-old chicks carrying FQ non-susceptible *E. coli*.

**Table 1:** Number of farms with *E. coli* and FQ non-susceptible *E. coli* in the environment (broiler house) before arrival of day-old chicks, specified for the different sampled locations within the house.

Sampled location	*E. coli* (*n* = 29)	FQ non-susceptible *E. coli* (*n* = 29)
Floor anteroom	11 (38%)	7 (24%)
Boots	10 (34%)	3 (10%)
Drinking cups	7 (24%)	3 (10%)
Feeding pans	15 (52%)	6 (21%)
Floor house	13 (45%)	7 (24%)
Drinking water	6 (21%)	1 (3%)
Nr of farms with at least one positive sample	26 (90%)	14 (48%)

**Table 2:** The number of farms that received day-old chicks with FQ non-susceptible *E. coli* in the gastrointestinal tract, the average percentage of chicks carrying FQ non-susceptibility, and the proportion of FQ non-susceptible *E. coli* (of total *E. coli*) at farm level, for each hatchery from which the flocks originated. The flocks of the 29 participating farms originated from 7 different hatcheries (A to G).

Hatchery	Number of flocks with FQ non-susceptible positive chicks (of total farms originating from same hatchery)	Percentage of chicks carrying FQ non-susceptibility (%)	Proportion FQ non-susceptible *E. coli* in FQ positive samples (%)
A	6/7	40.8	27.3
B	3/6	18.3	9.5
C	8/9	50.0	32.2
D	3/3	65.4	48.7
E	1/1	72.4	53.3
F	1/2	30.4	13.0
G	1/1	96.6	56.9
Total	23/29	43.9	28.3

### 3.3 FQ non-susceptibility during production cycle

**Figure 3:**
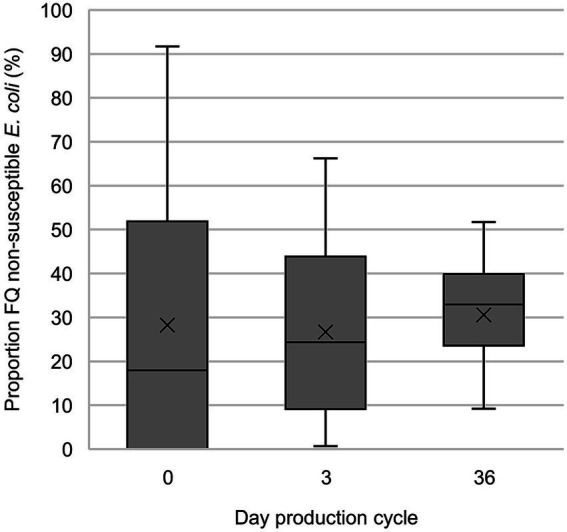
Proportion of fluoroquinolone (FQ) non-susceptible *E. coli* in the total *E. coli* population in fecal samples of broilers containing FQ non-susceptible *E. coli* (only taking into account samples that contained *E. coli*). On day 0, intestines and contents were sampled and on day 3 (±2) and 36 (± 3), cloacal swabs were investigated.

**Figure 4:**
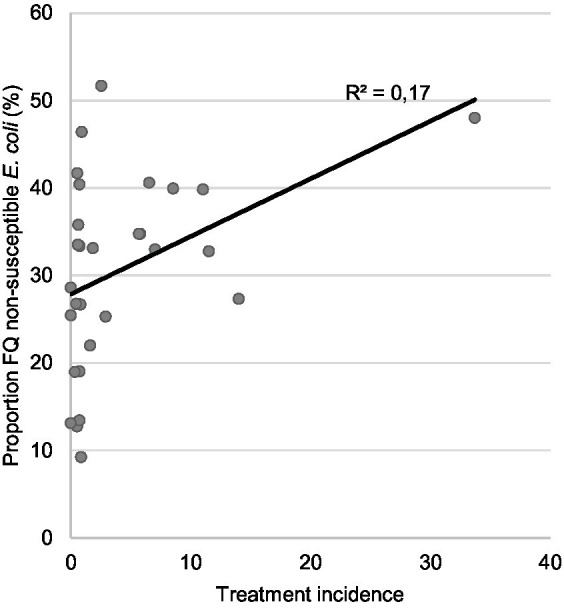
Scatter plot and corresponding regression line for the relationship between the dependant variable proportion of FQ non-susceptible *E. coli* at the end of the production cycle (day 36) and the independent variable total antimicrobial usage expressed in treatment incidence (TI) at the farm level.

On days 3 and 36, FQ non-susceptible *E. coli* were present at 100% of the farms (and in 94.0 and 99.4% of the broiler samples, respectively) (Figure 2). On days 0, 3 and 36, the proportion of FQ non-susceptible *E. coli* in the total *E. coli* population (only taking into account samples that contained *E. coli*) was 28.3%, 26.7, and 30.6%, respectively (Figure 3; Supplementary Table S6). The total TI of the production cycle was significantly associated with the proportion of FQ non-susceptible *E. coli* at the end of the production cycle (*r* = 0.42, 95% CI [0.06, 0.68], *p* = 0.03) (Figure 4). There was no significant difference in mean proportion of FQ non-susceptible *E. coli* at day 3 between the flocks treated with lincomycin and spectinomycin and the flocks that did not receive such treatment, with a mean difference of 4.87% (95% CI [−10.38, 20.10%], *p* = 0.52).

### 3.4 Multidrug-resistance

Of the 92 selected isolates (obtained from FQ supplemented plates) which underwent MIC determination, 63.6% was multidrug-resistant when applying ECOFF values (resistant against 3 or more antimicrobial classes) (Supplementary Table S7). Of the 66 isolates selected for WGS, nine isolates which were previously identified as *E. coli* by MALDI-TOF MS, were identified as *Escherichia fergusonii* based on WGS data (Supplementary Table S8). Of the remaining 57 *E. coli* isolates, 14 isolates originated from environmental samples, 22 isolates from day-old chicks and 21 isolates from broilers at day 36. The concordance between the detected genetic traits and the observed phenotypic resistance, known as the phenotypic-genotypic relationship, ranged from 93.0% for beta-lactam antibiotics to 100% for sulfonamides, trimethoprim and quinolones. About 80.7% of the isolates carried resistance genes against beta-lactam antimicrobials and 71.9% against aminoglycosides. Only one isolate, from a broiler at day 36, carried an ESBL gene (*bla*_CTX-M-55_). WGS detected genes and/or mutations associated with three or more classes of antibiotics in over 86% of the isolates (Supplementary Table S8).

### 3.5 Genetic traits FQ non-susceptibility

**Figure 5:**
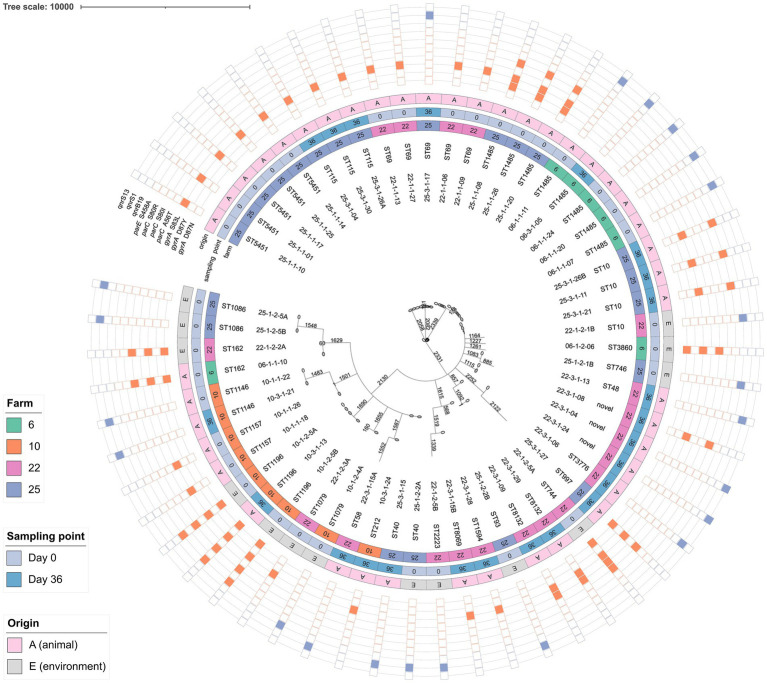
Phylogenetic tree of fluoroquinolone (FQ) non-susceptible *E. coli* based on core genome MLST. The FQ resistance genes and/or mutations are shown in the outer band: orange is the presence of QRDR mutations, purple is presence of *qnr* genes. Isolates from four farms were included, originating from sampling point day 0 and day 36. For every isolate the sampling name and sequence type are given. The letter A or E indicates whether the sample is taken from an animal (A) or the environment (E). The tree was constructed using the “MSTreeV2” method of GrapeTree. Branch lengths and the scale bar are expressed as the number of allelic differences.

Of the FQ non-susceptible isolates, 61.4% carried point mutations in the Quinolone Resistance-Determining Region (QRDR) and 38.6% carried *qnr* resistance genes (Figure 5). No other FQ resistance traits were detected. The proportions of *qnr* genes and QRDR mutations per sampling time point are listed in Table 3. All isolates with triple or quadruple QRDR mutations (*n =* 11) had ciprofloxacin MIC values > 4 mg/L (Supplementary Table S8). Of the 14 strains carrying *qnr* genes, two carried an additional AMR gene on the corresponding contig, being *bla*_CTX-M-55_ and *tet(A)* (Supplementary Table S8). All *E. fergusonii* isolates (*n* = 9), consisting of two clusters of identical isolates carried the *gyrA* S83L mutation. One cluster of *E. fergusonii* isolates (*n* = 7) additionally carried the *parE* I355T mutation.

**Table 3:** Proportion of *E. coli* isolates with QRDR mutations and *qnr* genes on day 0 (*n* = 34) and day 36 (*n* = 23).

Mutation/gene	Proportion of isolates on day 0 (%)	Proportion of isolates on day 36 (%)
*gyrA* D87N	17.6	8.7
*gyrA* D87Y	8.8	0
*gyrA* S83L	61.8	60.9
*parC* A56T	2.9	8.7
*parC* S80I	26.5	8.7
*parC* S80R	0	4.3
*parE* S458A	5.9	17.4
*qnrB19*	8.8	0
*qnrS1*	29.4	30.4
*qnrS13*	0	8.7

### 3.6 Virulence-associated genes

The 57 analyzed *E. coli* isolates carried on average 13 virulence-associated genes (VAGs) (SD ± 5.13). Of the 57 *E. coli* isolates, most common VAGs were *terC* (93.0%), *nlpI* (87.7%), *iss* (86.0%), *traT* (77.2%), *sitA* (75.4%), *hlyF* (64.9%), *csgA* (56.1%), *iutA* (56.1%), *iucC* (54.4%), and *lpfA* (52.6%). Other genes were present in less than 50% of the isolates (Supplementary Table S8).

### 3.7 Clustering

There was a large variation in the STs of FQ non-susceptible *E. coli*, both within and between farms (Figure 5). Three isolates were assigned to different novel STs (i.e., STs not yet introduced in the EnteroBase database). On farm 10 (ST1196) and 25 (ST40), FQ non-susceptible *E. coli* isolates collected on day 0 from the environment, showed identical cgMLST profiles and <5 pairwise SNP differences to isolates obtained from cloacal swabs of broilers on the same farm (Figure 5; Supplementary Table S6). In addition, on farms 6 (ST1485) and 10 (ST1146), isolates from day-old chicks clustered together with those from the broiler at the end of the production cycle. On farm 6, five ST1485 isolates exhibited ≤3 pairwise SNP differences, while on farm 10, two ST1146 isolates, for which SNP analysis was not possible due to the lack of a suitable reference genome, had identical cgMLST profiles. Moreover, isolates from different day-old chicks within the same farm clustered together, which was the case on farm 25 (three ST1485 isolates with 0 pairwise SNP differences), farm 22 (three ST69 isolates with 0–4 pairwise SNP differences), and farm 6 (four ST1485 isolates with 1–4 pairwise SNP differences) (Supplementary Table S9). For ST5451, five isolates collected from chicks on day 0 shared identical cgMLST profiles. However, no suitable reference genome was available on EnteroBase, and SNP analysis was not performed.

## 4 Discussion

FQ are classified as critically important antimicrobials by both the World Health Organization and the World Organization of Animal Health (33, 34). To reduce FQ resistance prevalence through targeted interventions, more insight into the role of the environment, day-old chicks and AMU in the spread of FQ resistance in the broiler industry is required. Our study revealed that *E. coli* isolates with non-wild type FQ phenotype were present on all sampled broiler farms at day 3 until the end of the production cycle. The proportion of FQ non-susceptible *E. coli* stayed relatively stable at around 30% from day of hatch till day 36. Notably, these non-susceptible strains persisted even on 29 farms where no FQ were used, suggesting the influence of other contributing factors. Our study showed that FQ non-susceptible *E. coli* are often multidrug-resistant strains, as also previously published (7, 35). This could explain the persistence of FQ non-susceptibility through co-selection by the use of other antimicrobials (36–39). This hypothesis is supported by our WGS results, showing a high number of isolates carrying resistance genes against aminoglycosides and beta-lactam antibiotics. These antimicrobial classes are frequently used in the broiler industry, including in the farms in our study. In particular, aminoglycoside usage was common, as 20 farms in our study started their production cycle with a treatment of lincomycin combined with spectinomycin. Additionally, one farm showed substantially higher antibiotic usage compared to the others. After careful review, this observation was retained, as there was no indication of an error and it accurately reflected the variability in antibiotic use across farms. All in all, co-selection could account for the significant, though weak, positive correlation we found between the total TI during the production cycle and the proportion of FQ non-susceptible *E. coli* at the end of the cycle, indicating that reducing only FQ use is insufficient to limit resistance selection pressure and a broader reduction in total AMU is probably warranted. In addition, two strains harboring a *qnr* gene carried an additional AMR gene on the corresponding contig, being *bla*_CTX-M-55_ and a *tet(A)* gene. Both genes are commonly plasmid-associated, suggesting possible co-selection of AMR genes (40, 41). However, as only short-read sequencing data is available, plasmid sequences are likely fragmented and additional co-localizations of *qnrS* with other AMR genes may have been missed. Further studies are needed to determine the effect of co-selection and antibiotic treatment strategies on the emergence of multidrug resistance in *E. coli*.

However, AMU accounted for only a small portion of the variability in the proportion of FQ non-susceptible *E. coli*, indicating that other factors also have an impact on the occurrence of FQ resistance. On the three farms that did not use any antimicrobials, FQ non-susceptible *E. coli* were found in (almost) all sampled broilers at day 3 and 36. The fact that even without any antimicrobial selection pressure, the FQ non-susceptible *E. coli* strains can persist in the broiler population, suggests that the fitness costs of the present FQ resistance mechanisms are low or even non-existent (42, 43). The study by Marcusson et al. (42) showed that an initial mutation (mostly *gyrA*) had a fitness cost, but later compensatory *parC* mutations could confer an increase in both resistance and fitness. This was later confirmed by the study of Machuca et al. (43), where an enhanced bacterial fitness was observed when there was a combination of mutations in the QRDR region or in the presence of only the *qnrS1* gene. In our study, three of the four clusters of persistent isolates carried only a *qnrS1* gene, and the other cluster carried a combination of QRDR mutations, including mutations in *gyrA* and *parC*. Also, the FQ non-susceptible *E. coli* isolates carried on average 13 virulence-associated genes. VAGs are commonly used to differentiate pathogenic *E. coli* strains from commensal ones. However, making a clear distinction remains challenging due to the extensive genetic diversity within the *E. coli* species (44–46). Ovi et al. (46) identified 10 key VAGs in avian pathogenic *E. coli* isolates compared to non-pathogenic strains, serving three main functional roles, being secretion-related functions (*cvaC, episomal ompT, hlyF*), iron acquisition (*iroN, iutA, iucD*), and host attachment, invasion, and survival (*tsh, papG, hlyF, iss*). However, other studies reported different genes being common in APEC strains, like *traT, sitA, csgA, lpfA, iucC* (44, 45, 47, 48). Notably, many of these possible pathogenic related VAGs were also present in the FQ non-susceptible isolates from our study, being *iss*, *traT, sitA, hlyF, csgA, iutA, iucC, lpfA*, and *cvaC*.

However, commensal *E. coli* strains can also harbor VAGs which can offer a competitive advantage in the gut microbiota (44). These genes may not necessarily confer pathogenicity but can improve survival and competitiveness in various ecological niches (49). Pakbin et al. (48) reported the presence of several VAGs from human enteric *E. coli* pathotypes which play a role in survival influencing colonization (e.g., *csgA*), fitness (e.g., *iutA, kpsMTII, fyuA*), toxin production (e.g., *astA, hlyE*), and effector functions promoting adherence and biofilm formation (e.g., *espB, espA, espF, espJ*). The presence of these VAGs could enhance survival in both gut and environmental settings (44, 48). For example, *sitA* is linked to increased resistance to oxidative stress, and iron acquisition systems is considered being beneficial for environmental persistence (44). Moreover, commensal isolates may act as reservoirs for transferable VAGs (50), facilitating the spread of virulence traits within microbial communities. The FQ non-susceptible *E. coli* isolates in this study carried on average 13 VAGs, which does not necessarily indicate pathogenic potential. However, the presence of these genes could contribute to enhanced fitness, colonization, and survival in both host-associated and external environments (44, 45, 47, 48, 51), which could be an explanation for their survival within the broiler population. Further research is needed to investigate the fitness of these FQ non-susceptible strains and to link specific resistance mechanisms and virulence-associated genes to their potential fitness effects.

Furthermore, according to our cgMLST clustering, FQ non-susceptible *E. coli* isolates obtained from both day-old chicks and the environment present at day 0 can persist in chicks throughout the whole production cycle, indicating that these strains play a role in the persistence of FQ resistance in broilers. Regarding environmental dissemination, previous studies have already described the persistence of quinolone resistant *E. coli* in the broiler production environment and hypothesized that the occurrence in broilers may be influenced by on-farm recirculation (15, 18, 52, 53). Our findings support this hypothesis, as in 45% of the farms, FQ non-susceptible *E. coli* were found in the house environment before the animals even entered the stable and were able to persist until the end of the production cycle. In Belgium it is common practice to clean and disinfect the house between production cycles, although the used methods can vary in effectiveness (22, 54). The high prevalence of FQ non-susceptible *E. coli* in the environment highlights the critical need for performing the cleaning and disinfection more thoroughly to prevent cross-contamination between production rounds. However, contamination could also occur through the introduction of materials into the house after cleaning and disinfection, such as through feed (55), or due to inadequate biosecurity measures during preparation of the house (5). A multifactorial approach is therefore needed, improving biosecurity measures and management (56).

Yet, our study also clearly indicated that biosecurity measures on the farm level alone will not be sufficient to stop the introduction of FQ non-susceptible *E. coli*. In 79.3% of the farms, day-old chicks arrived with FQ non-susceptible *E. coli* in their gastrointestinal tracts, confirming previous reports of vertical transmission of these isolates throughout the broiler production pyramid (14–17). In our study, the isolates from day-old chicks within the farm clustered together for three out of four farms, from which the isolates were analyzed through WGS. This suggests that a batch of day-old chicks may acquire FQ non-susceptible *E. coli* from a common source or there is clonal spread within a batch of chicks, which could be facilitated through the high density in which the chicks are being held in hatcheries and during transport. A previous study showed the potential entry of *Enterobacterales* from the parent flock into the hatchery, where ESBL-producing *E. coli* were detected on 0.9% of egg surfaces and in the hatchery environment, however, no positive isolates were found in hatchlings (12). The presence of resistant isolates on eggshells of broiler hatching eggs was also detected by Mezhoud et al. (57). A different study focused specifically on quinolone-resistant *E. coli* and conducted longitudinal sampling across all levels of the production pyramid and highlighted the role of environmental recirculation (15). To better understand how and when these chicks are colonized with FQ non-susceptible *E. coli* isolates, further research is needed, particularly investigating the role of the hatchery and (grand)parent stock.

Regarding underlying FQ resistance mechanisms, our study found a substantial number of isolates carrying PMQR genes. Previous studies on FQ dissemination in the broiler production chain did not identify PMQR genes in broiler *E. coli* isolates (14–16), likely due to differences in screening methods. For example, these studies selected isolates based on nalidixic acid resistance (>16 mg/L), but *E. coli* isolates carrying PMQR genes often exhibit only a minor reduction in susceptibility to nalidixic acid (58). Our findings corroborate this, as the isolates carrying PMQR genes exhibited lower MIC values to FQ compared to those with QRDR mutations. Although Myrenås et al. (17) specifically selected FQ isolates with MIC profiles indicative of the presence of PMQR genes (ciprofloxacin MIC ≥ 0.06 mg/L combined with nalidixic acid MICs 4–32 mg/L), they could identify only one isolate carrying a *qnrS* gene. De Koster et al. (59) found PMQR genes (*qnrS1*) in 8.9% of the ciprofloxacin resistant *E. coli* broiler isolates, using selective plates supplemented with ciprofloxacin (2 mg/L) according to the clinical breakpoint for the detection of FQ isolates. These isolates carried PMQR genes in combination with QRDR mutations. In the study of Temmerman et al. (35), which only included avian pathogenic *E. coli* isolates collected from colibacillosis outbreaks, PMQR associated genes were found in 18% of non-wild type FQ resistant isolates, of which *qnrS* was detected in 14% and *qnrB* in 4% of the isolates. These different study outcomes suggest that the screening method might play a role in the detection of PMQR genes. Due to the specific selection of isolates in our study, our prevalence rates are not directly comparable to those of other studies.

Additionally, other *Enterobacterales* may contribute to the circulation of AMR in the environment and animals. After a pre-selection for *E. coli* and using WGS, we identified nine FQ non-susceptible *E. fergusonii* isolates, which can be explained by the fact that the MALDI-TOF database only contained one *E. fergusonii* entry and the related software confirmed it as non-distinguishable from *E. coli* due to the close relatedness. Previous studies have confirmed the potential of *E. fergusonii* as a reservoir for AMR (60–63). Ferreira et al. (64) described *E. fergusonii* isolates as being multidrug-resistant and also carrying the *qnr*B19 gene. In our study, only QRDR mutations were found in *E. fergusonii* isolates, but further research is needed to determine the role of *E. fergusonii* and other *Enterobacterales* in the dissemination of FQ non-susceptibility.

Regarding the screening of FQ residues, the FQ usage on one farm was confirmed through residue detection in collected feather samples well above the LOQ (50–600 ng/g feather). On another farm, very low (1.2–3.7 ng/g feather) flumequine residue concentrations were detected, despite no registered usage. A recent study showed that broilers treated with flumequine in the first 3 days of life had residue levels in feathers ranging from 3.4 to 15 ng/g feather on day 38 of the production cycle (23). The low residue levels in the current study could suggest unreported use at the beginning of the production cycle or uptake of residues from the surroundings which could originate from usage in a previous cycle, as FQ have been described to be notoriously stable in the environment (65, 66).

## 5 Conclusion

The continued presence of FQ non-susceptible *E. coli* on broiler farms is likely due to both historical contamination at the farm level and a continuous influx along the production chain. AMU contributes to the continued presence of FQ non-susceptible *E. coli* in broiler farms, but accounted for only a small proportion of the variability in FQ non-susceptibility in the currently investigated farms. The role of certain virulence-associated genes in the persistence of FQ non-susceptible *E. coli* in broiler farms deserves more in-depth research. To effectively combat this issue, efforts must focus on preventing the transmission of resistant bacteria, both at the farm level and throughout the broiler production chain, together with further reductions in AMU.

## Data Availability

The data presented in the study are deposited in the National Center for Biotechnology Information (NCBI) under BioProject number PRJNA1192038. The accession numbers can be found in Supplementary Table S8.
