# Supplementary material for: Mapping the spread of fluoroquinolone resistance: continued presence of non-susceptible Escherichia coli in broilers
Source: Front Vet Sci. 2025 Sep 19;12:1610997. doi: 10.3389/fvets.2025.1610997 (PMC12491820; doi:10.3389/fvets.2025.1610997)
Supplement: Supplementary file 1 [file Table_1.docx]

Supplementary Material

**Table S1.** Methodology processing collected samples for bacteriological analysis, WGS and SNP analysis.

| Reference genomes used in the SNP analysis | |
| --- | --- |
| Sequence type | Reference genome accession number |
| ST428 | NZ_CP049101.1 |
| ST10 | NZ_CP038791.1 |
| ST117 | NZ_CP029164.1 |
| ST7852 | NZ_CP083638.1 |

**Table S2.** Farm characteristics and sampling dates 29 broiler farms

| Farm_ID | Day 0 | Day 3 ± 2 days | Day 36 ± 3 days | Number of stables | Capacity_sampled_house | Hatchery_code | Age_parentstock |
| --- | --- | --- | --- | --- | --- | --- | --- |
| 1 | 8-10-2020 | 13-10-2020 | 12-11-2020 | 3 | 32040 | A | 32 |
| 2 | 7-6-2021 | 11-6-2021 | 15-7-2021 | 8 | 45000 | B | 53 |
| 3 | 29-9-2021 | 2-10-2021 | 7-11-2021 | 2 | 18500 | C | 38 |
| 4 | 3-2-2021 | 6-2-2021 | 10-3-2021 | 2 | 37000 | D | 58 |
| 5 | 21-6-2021 | 26-6-2021 | 29-7-2021 | 2 | 48500 | C | 48 |
| 6 | 29-6-2021 | 2-7-2021 | 5-8-2021 | 1 | 23000 | E | 27 |
| 7 | 6-10-2020 | 9-10-2020 | 10-11-2020 | 1 | 27990 | F | NA |
| 8 | 3-3-2021 | 6-3-2021 | 9-4-2021 | 2 | 27810 | B | 95 |
| 9 | 9-2-2021 | 12-3-2021 | 16-3-2021 | 2 | 43750 | D | 59 |
| 10 | 15-12-2020 | 18-12-2020 | 19-1-2021 | 2 | 42500 | C | 38 |
| 11 | 21-1-2021 | 24-1-2021 | 25-2-2021 | 4 | 64000 | A | 54 |
| 12 | 18-2-2021 | 21-2-2021 | 23-3-2021 | 2 | 42250 | B | 46 |
| 13 | 19-2-2021 | 23-2-2021 | 26-3-2021 | 1 | 18000 | C | 51 |
| 14 | 4-6-2021 | 8-6-2021 | 13-7-2021 | 5 | 47000 | A | 45 |
| 15 | 24-9-2021 | 26-9-2021 | 2-11-2021 | 1 | 18500 | C | 37 |
| 16 | 4-5-2021 | 7-5-2021 | 10-6-2021 | 6 | 25000 | D | 42 |
| 17 | 13-11-2020 | 16-11-2020 | 21-12-2020 | 3 | 23400 | B | 50 |
| 18 | 22-4-2021 | 25-4-2021 | 26-5-2021 | 2 | 26640 | B | 53 |
| 19 | 25-6-2021 | 28-6-2021 | 3-8-2021 | 4 | 44100 | A | 48 |
| 20 | 28-7-2021 | 2-8-2021 | 2-9-2021 | 3 | 17000 | A | 49 |
| 22 | 18-10-2021 | 21-10-2021 | 23-11-2021 | 1 | 24000 | C | 55 |
| 21 | 19-7-2021 | 22-7-2021 | 27-8-2021 | 2 | 30000 | A | 40 |
| 23 | 26-8-2021 | 30-8-2021 | 4-10-2021 | 4 | 80000 | C | 48 |
| 24 | 7-10-2021 | 11-10-2021 | 11-11-2021 | 3 | 56000 | C | 34 |
| 25 | 15-10-2021 | 19-10-2021 | 22-11-2021 | 1 | 42500 | A | 63 |
| 26 | 12-11-2021 | 15-11-2021 | 20-12-2021 | 2 | 40000 | B | 58 |
| 27 | 27-9-2021 | 30-9-2021 | 4-11-2021 | 1 | 14400 | G | 46 |
| 28 | 23-9-2021 | 25-9-2021 | 1-11-2021 | 1 | 50000 | C | 60 |
| 29 | 28-9-2021 | 1-10-2021 | 5-11-2021 | 3 | 19260 | F | 40 |

**Table S3.** Antimicrobial usage farms during sampled production cycle expressed in treatment incidence (TI). The TI is equal to the number of days a broiler was treated with antimicrobials in a theoretical period of 100 days, which can also be interpreted as the percentage of the time a broiler was treated with antimicrobials during its life.

| Farm_ID | TI_total | TI_Amoxicillin | TI_Doxycycline | TI_Flumequine | TI_Lincomycin | TI_Lincomycin_Spectinomycin | TI_Phenoxymethylpenicillin | TI_Trimethoprim + sulfadiazinum | TI_Tylosin |
| --- | --- | --- | --- | --- | --- | --- | --- | --- | --- |
| 1 | 14,00591905 | 0 | 0 | 0 | 0 | 2,969502 | 0 | 11,03642 | 0 |
| 2 | 1,840372536 | 0 | 0 | 0 | 0 | 0,649123 | 0 | 0 | 1,19125 |
| 3 | 0 | 0 | 0 | 0 | 0 | 0 | 0 | 0 | 0 |
| 4 | 2,549525716 | 0 | 0 | 0 | 0 | 0,642857 | 0 | 0 | 1,906669 |
| 5 | 6,542246566 | 0 | 0 | 0 | 0 | 0,596256 | 5,611056 | 0 | 0,334934 |
| 6 | 5,802904354 | 0 | 0 | 0 | 0 | 0 | 0 | 0 | 5,802904 |
| 7 | 33,71565484 | 17,8 | 0 | 0 | 4,700302 | 1,121727 | 0 | 10,10666 | 0 |
| 8 | 1,62106251 | 0 | 1,619743 | 0 | 0 | 0 | 0 | 0 | 0,00132 |
| 9 | 0,719866163 | 0 | 0 | 0 | 0 | 0,717649 | 0 | 0 | 0,002217 |
| 10 | 0,820840336 | 0 | 0 | 0 | 0 | 0,82084 | 0 | 0 | 0 |
| 11 | 0,743303571 | 0 | 0 | 0 | 0 | 0,743304 | 0 | 0 | 0 |
| 12 | 11,03432977 | 0 | 0 | 10,24616 | 0 | 0,788166 | 0 | 0 | 0 |
| 13 | 0,872142857 | 0 | 0 | 0 | 0 | 0,872143 | 0 | 0 | 0 |
| 14 | 0,599509002 | 0 | 0 | 0 | 0 | 0,599509 | 0 | 0 | 0 |
| 15 | 7,036999307 | 6,0 | 0 | 0 | 0 | 0,999231 | 0 | 0 | 0 |
| 16 | 8,541875209 | 0 | 7,207207 | 0 | 0 | 0 | 0 | 0 | 1,334668 |
| 17 | 0,61791498 | 0 | 0 | 0 | 0 | 0,617915 | 0 | 0 | 0 |
| 18 | 0,921156723 | 0 | 0 | 0 | 0 | 0,919118 | 0 | 0 | 0,002039 |
| 19 | 2,907145764 | 0 | 2,907146 | 0 | 0 | 0 | 0 | 0 | 0 |
| 20 | 0,734558824 | 0 | 0 | 0 | 0 | 0,734559 | 0 | 0 | 0 |
| 22 | 11,51281007 | 10,1 | 0 | 0 | 0 | 0 | 0 | 0 | 1,428898 |
| 21 | 0 | 0 | 0 | 0 | 0 | 0 | 0 | 0 | 0 |
| 23 | 0,352646803 | 0 | 0 | 0 | 0 | 0,352212 | 0 | 0 | 0,000435 |
| 24 | 0,560663265 | 0 | 0 | 0 | 0 | 0,560663 | 0 | 0 | 0 |
| 25 | 0,515479876 | 0 | 0 | 0 | 0 | 0,51548 | 0 | 0 | 0 |
| 26 | 0,722960526 | 0 | 0 | 0 | 0 | 0,722961 | 0 | 0 | 0 |
| 27 | 0 | 0 | 0 | 0 | 0 | 0 | 0 | 0 | 0 |
| 28 | 5,69174359 | 0 | 5,128205 | 0 | 0 | 0,563538 | 0 | 0 | 0 |
| 29 | 0,454992622 | 0 | 0 | 0 | 0 | 0,454993 | 0 | 0 | 0 |

**Table S4.** Residue concentrations of enrofloxacin, ciprofloxacin, and flumequine in feathers of broilers sampled at the end of the production cycle by using a UPHLC-MS/MS method.

Table is available in supplementary excel file.

**Table S5.** Presence *E. coli* and FQ non-susceptible *E. coli* in environmental samples from broiler houses.

|  | | Floor anteroom | | Boots | | Drinking cups | | Feeding pan | | Floor house | | Drinking water | |
| --- | --- | --- | --- | --- | --- | --- | --- | --- | --- | --- | --- | --- | --- |
| Farm_ID | Date_sampling | *E.coli* | *FQ E.coli* | *E.coli* | *FQ E.coli* | *E.coli* | *FQ E.coli* | *E.coli* | *_FQ E.coli* | *E.coli* | *FQ E.coli* | *E.coli* | *FQ E.coli* |
| 1 | 8-10-2020 | + | + | - | - | - | - | + | + | - | - | - | - |
| 2 | 7-6-2021 | - | - | - | - | - | - | + | - | - | - | + | - |
| 3 | 29-9-2021 | - | - | - | - | - | - | + | - | - | - | - | - |
| 4 | 3-2-2021 | - | - | - | - | - | - | - | - | - | - | - | - |
| 5 | 21-6-2021 | + | + | - | - | - | - | + | - | + | + | - | - |
| 6 | 29-6-2021 | - | - | - | - | - | - | - | - | - | - | + | + |
| 7 | 6-10-2020 | - | - | + | - | + | - | - | - | - | - | - | - |
| 8 | 3-3-2021 | - | - | - | - | - | - | + | - | - | - | - | - |
| 9 | 9-2-2021 | - | - | - | - | - | - | - | - | - | - | - | - |
| 10 | 15-12-2020 | - | - | - | - | - | - | + | + | + | + | - | - |
| 11 | 21-1-2021 | - | - | - | - | - | - | - | - | - | - | - | - |
| 12 | 18-2-2021 | - | - | - | - | - | - | - | - | + | - | - | - |
| 13 | 19-2-2021 | + | - | + | + | - | - | + | - | + | - | - | - |
| 14 | 4-6-2021 | - | - | - | - | - | - | + | - | - | - | - | - |
| 15 | 24-9-2021 | + | + | + | - | + | + | - | - | + | - | - | - |
| 16 | 4-5-2021 | + | - | + | - | - | - | - | - | + | + | + | - |
| 17 | 13-11-2020 | - | - | + | - | - | - | - | - | - | - | - | - |
| 18 | 22-4-2021 | - | - | - | - | + | - | + | + | + | - | - | - |
| 19 | 25-6-2021 | - | - | + | - | - | - | - | - | - | - | - | - |
| 20 | 28-7-2021 | - | - | - | - | - | - | + | - | - | - | + | - |
| 21 | 19-7-2021 | + | + | + | - | + | + | + | + | + | + | - | - |
| 22 | 18-10-2021 | + | + | + | + | + | + | + | + | + | + | - | - |
| 23 | 26-8-2021 | - | - | - | - | - | - | - | - | + | - | - | - |
| 24 | 7-10-2021 | + | - | + | - | + | - | + | - | + | + | + | - |
| 25 | 15-10-2021 | + | + | + | + | + | - | - | - | + | + | - | - |
| 26 | 12-11-2021 | - | - | - | - | - | - | - | - | - | - | + | - |
| 27 | 27-9-2021 | - | - | - | - | - | - | + | + | - | - | - | - |
| 28 | 23-9-2021 | + | + | - | - | - | - | + | - | + | - | - | - |
| 29 | 28-9-2021 | + | - | - | - | - | - | - | - | - | - | - | - |

**Table S6.** Fluoroquinolone (FQ) resistance measured during the different sampling moments of the production cycle (day 0, 3 and 36). Given are the percentage of FQ non-susceptible *E. coli* positive samples and the average proportion of FQ non-susceptible *E. coli.*

Table is available in supplementary excel file .

**Table S7.** Percentage of FQ non-susceptible *E. coli* resistant against total number of antibiotic groups according to ECOFF wild type cut-off, on a total of 93 isolates.

| Number of antibiotic groups | Number of ECOFF resistant strains |
| --- | --- |
| 0 | - |
| 1 | 11 (11.8%) |
| 2 | 22 (23.7%) |
| 3 | 17 (18.3%) |
| 4 | 27 (29.0%) |
| 5 | 15 (16.1%) |
| 6 | - |
| 7 | 1 (1.1%) |

**Table S8.** The minimum inhibitory concentrations of the selected 92 isolates. Of which from 66 isolates the fluoroquinolone non-susceptible *E.coli* sequences are given together with their genetic characteristics.

Table is available in supplementary excel file.

**Table S9.** SNP distance matrices of isolates belonging to different clusters according to cgMLST.

**ST69**

|  | **22_1_1_09** | **22_1_1_06** | **22_1_1_13** | **22_1_1_27** | **29_2_1_07** | **23_1_1_07** | **23_1_1_23** | **23_1_1_20** | **23_1_1_11** | **23_1_1_21** | **23_1_1_19** | **23_1_1_08** | **23_1_1_22** | **25_3_1_17** | **23_1_1_26** |
| --- | --- | --- | --- | --- | --- | --- | --- | --- | --- | --- | --- | --- | --- | --- | --- |
| **22_1_1_09** | 0 | 2 | 4 | 6 | 864 | 8429 | 8426 | 8425 | 8430 | 8428 | 8411 | 8430 | 8430 | 7448 | 7387 |
| **22_1_1_06** | 2 | 0 | 3 | 5 | 854 | 8408 | 8404 | 8405 | 8408 | 8407 | 8394 | 8408 | 8408 | 7421 | 7369 |
| **22_1_1_13** | 4 | 3 | 0 | 2 | 857 | 8419 | 8416 | 8415 | 8420 | 8418 | 8400 | 8420 | 8420 | 7440 | 7383 |
| **22_1_1_27** | 6 | 5 | 2 | 0 | 860 | 8418 | 8415 | 8414 | 8419 | 8417 | 8399 | 8419 | 8419 | 7434 | 7375 |
| **29_2_1_07** | 864 | 854 | 857 | 860 | 0 | 8087 | 8083 | 8083 | 8087 | 8086 | 8074 | 8087 | 8087 | 7029 | 7139 |
| **23_1_1_07** | 8429 | 8408 | 8419 | 8418 | 8087 | 0 | 0 | 0 | 0 | 0 | 0 | 0 | 0 | 3684 | 2912 |
| **23_1_1_23** | 8426 | 8404 | 8416 | 8415 | 8083 | 0 | 0 | 0 | 0 | 0 | 0 | 0 | 0 | 3682 | 2912 |
| **23_1_1_20** | 8425 | 8405 | 8415 | 8414 | 8083 | 0 | 0 | 0 | 0 | 0 | 0 | 0 | 0 | 3681 | 2910 |
| **23_1_1_11** | 8430 | 8408 | 8420 | 8419 | 8087 | 0 | 0 | 0 | 0 | 0 | 0 | 0 | 0 | 3684 | 2912 |
| **23_1_1_21** | 8428 | 8407 | 8418 | 8417 | 8086 | 0 | 0 | 0 | 0 | 0 | 0 | 0 | 0 | 3684 | 2912 |
| **23_1_1_19** | 8411 | 8394 | 8400 | 8399 | 8074 | 0 | 0 | 0 | 0 | 0 | 0 | 0 | 0 | 3672 | 2904 |
| **23_1_1_08** | 8430 | 8408 | 8420 | 8419 | 8087 | 0 | 0 | 0 | 0 | 0 | 0 | 0 | 0 | 3684 | 2912 |
| **23_1_1_22** | 8430 | 8408 | 8420 | 8419 | 8087 | 0 | 0 | 0 | 0 | 0 | 0 | 0 | 0 | 3683 | 2910 |
| **25_3_1_17** | 7448 | 7421 | 7440 | 7434 | 7029 | 3684 | 3682 | 3681 | 3684 | 3684 | 3672 | 3684 | 3683 | 0 | 2736 |
| **23_1_1_26** | 7387 | 7369 | 7383 | 7375 | 7139 | 2912 | 2912 | 2910 | 2912 | 2912 | 2904 | 2912 | 2910 | 2736 | 0 |

**ST1196**

|  | **10_3_1_13** | **10_1_2_5A** | **10_1_2_5B** |
| --- | --- | --- | --- |
| **10_4_1_13** | 0 | 2 | 2 |
| **10_1_2_5A** | 2 | 0 | 2 |
| **10_1_2_5B** | 2 | 2 | 0 |

**ST1485**

|  | **23_3_1_08** | **25_1_1_26** | **25_1_1_08** | **25_1_1_20** | **29_3_1_14** | **06_1_1_11** | **06_1_1_07** | **06_1_1_24** | **06_1_1_20** | **06_3_1_05** |
| --- | --- | --- | --- | --- | --- | --- | --- | --- | --- | --- |
| **23_3_1_08** | 0 | 103 | 103 | 103 | 5852 | 5878 | 5874 | 5876 | 5858 | 5862 |
| **25_1_1_26** | 103 | 0 | 0 | 0 | 5897 | 5921 | 5915 | 5919 | 5901 | 5905 |
| **25_1_1_08** | 103 | 0 | 0 | 0 | 5898 | 5922 | 5916 | 5920 | 5902 | 5906 |
| **25_1_1_20** | 103 | 0 | 0 | 0 | 5898 | 5922 | 5916 | 5920 | 5902 | 5906 |
| **29_3_1_14** | 5852 | 5897 | 5898 | 5898 | 0 | 111 | 107 | 109 | 108 | 108 |
| **06_1_1_11** | 5878 | 5921 | 5922 | 5922 | 111 | 0 | 4 | 4 | 3 | 3 |
| **06_1_1_07** | 5874 | 5915 | 5916 | 5916 | 107 | 4 | 0 | 2 | 1 | 1 |
| **06_1_1_24** | 5876 | 5919 | 5920 | 5920 | 109 | 4 | 2 | 0 | 1 | 1 |
| **06_1_1_20** | 5858 | 5901 | 5902 | 5902 | 108 | 3 | 1 | 1 | 0 | 0 |
| **06_3_1_05** | 5862 | 5905 | 5906 | 5906 | 108 | 3 | 1 | 1 | 0 | 0 |

**ST1146** No suitable reference found in original analysis, but strains appear to be identical.
